# Da-Yuan-Yin decoction polyphenol fraction attenuates acute lung injury induced by lipopolysaccharide

**DOI:** 10.1080/13880209.2023.2166085

**Published:** 2023-01-18

**Authors:** Lengqiu Guo, Yun Yang, Jie Yuan, Huiling Ren, Xiaolei Huang, Meng Li, Long Xia, Xiaogang Jiang, Daofeng Chen, Jian Zhang

**Affiliations:** a Suzhou Vocational Health College, Suzhou, China; b College of Pharmaceutical Science, Soochow University, Suzhou, China; c Anhui Institutes for Food and Drug Control, Hefei, China; d School of Pharmacy, Fudan University, Shanghai, China

**Keywords:** Complement, oxidase, inflammatory factors, NF-κB, IKK, network pharmacology

## Abstract

**Context:**

Da-Yuan-Yin is a Chinese traditional prescription.

**Objective:**

This study explores the therapeutic effects of the Da-Yuan-Yin decoction polyphenol fraction (DYY-4) on acute lung injury (ALI) in mice induced by lipopolysaccharide (LPS).

**Materials and methods:**

The mice (*n* = 10) were orally administrated with DYY-4 (15, 30, and 60 mg/kg) or DXM (5 mg/kg), half an hour after LPS (2 mg/kg) instilled intratracheally. The protein content and the levels of inflammatory factors, the levels of complements, the mRNA expression of inducible nitric oxide synthase (iNOS) and cyclooxygenase-2 (COX-2), the level of myeloperoxidase (MPO) and superoxide dismutase (SOD), the expression of the IkB kinase (IKK) and nuclear factor-kappa B (NF-κB), the lung wet-to-dry weight (W/D) ratio and lung tissue were evaluated, 24 h after LPS challenge. Network pharmacology predicted potential targets.

**Results:**

DYY-4 (30, 60 mg/kg, *p* < 0.01, *p* < 0.01) decreased the lung W/D ratio, total protein concentration, the levels of C3, C3c and C5a, the levels of TNF-α, IL-6, and IL-1β, while increased the levels of IL-4 and IL-10. DYY-4 (60 mg/kg) decreased the levels of C5aR1, C5b-9 and COX-2 mRNA (*p* < 0.05), the levels of MPO and iNOS mRNA, the activation of the IKK/NF-κB pathway (*p* < 0.01), and increased the levels of IL-13 and SOD (*p* < 0.01). DYY-4 (60 mg/kg) relieved the lung tissue pathological changes and reduced the C3c deposition.

**Discussion and conclusions:**

Network pharmacology combined with animal experiments revealed the targets of DYY-4 alleviating ALI.

## Introduction

Acute lung injury (ALI) originates from many direct and indirect injury factors. Its severe form is acute respiratory distress syndrome (ARDS). The main pathological features of ALI are diffuse lung cell injury, inflammatory cell infiltration in lung tissue and pulmonary edoema caused by pulmonary vascular injury (Shi et al. 2014; Tian et al. 2019). The pathogenesis of ALI is complicated. Various immunomodulators that play critical roles in ALI, such as NO, iNOS, COX-2, IL-1β, IL-6, and TNF-α are regulated by nuclear factor-kappa B (NF-κB) and active IκBα kinase (IKK) signalling pathways (Qiao et al. 2013; Pei et al. 2019; Um et al. 2020). The pro-inflammatory factors such as IL-1β, IL-6 and TNF-α appear in the early phase of inflammatory response and aggravate lung injury (Pei et al. 2019). The anti-inflammatory factors such as IL-4, IL-10 and IL-13 can alleviate lung injury (Wu et al. 2009; Wang et al. 2016). The activation of the complement system and active complement fragments may exacerbate lung injury (Guo and Ward 2005; Sarma and Ward 2011; Bosmann and Ward 2012). In the clinic, the elevation of complement 3a (C3a) and complement 5a (C5a) in bronchoalveolar lavage fluid (BALF) are used as the diagnostic indicator of ALI (Guo and Ward 2005; Bosmann and Ward 2012; Wu et al. 2021). Additionally, oxidative damage can exacerbate lung damage.

Da-Yuan-Yin (DYY) developed by Youke Wu, who was a famous doctor in the early Qing Dynasties, in Ancient China, is used to treat various types of inflammation (Li et al. 2010; Ren et al. 2015; Wei et al. 2017). As shown in Table 1, Da-Yuan-Yin consists of seven traditional Chinese medicines (Li et al. 2010; Ren et al. 2015; Wei et al. 2017). Da-Yuan-Yin decoction is the basic prescription for treating patients with Coronavirus disease 2019 (COVID-19) in China (Headquarters for prevention and control of infected pneumonia in COVID-19, Hubei Province, China, 2020).

**Table 1.** The components of Da-Yuan-Yin decoction.

Synergy	Sovereign drug	Minister drug	Minister drug	Assistant drug	Assistant drug	Assistant drug	Envoy drug
Traditional Chinese medicine	*Arecae semen*	*Magnoliae officinalis cortex*	*Tsaoko fructus*	*Anemarrhenae rhizoma*	*Paeoniae radix alba*	*Scutellariae radix*	*Glycyrrhizae radix et rhizoma*
Chinese Pinyin	Binglang	Houpo	Caoguo	Zhimu	Baishao	Huangqin	Gancao
Plant origin	*Areca catechu* L.	*Magnolia officinalis* Rehd. et Wils	*Amomum tsao-ko*	*Anemarrhena asphodeloides* Bge.	*Paeonia ladiflora* Pall.	*Scutellaria baicalensis* Georgi	*Glycyrrhiza uralensis* Fisch.
Abbre*via*tion	BL	HP	CG	ZM	BS	HQ	GC

During the last decade, we performed several studies on Da-Yuan-Yin (Ren et al. 2015; Wei et al. 2017). Da-Yuan-Yin decoction showed a significant antipyretic effect on lipopolysaccharide (LPS) induced febrile response in rats (Ren et al. 2015). The main chemical compositions of Da-Yuan-Yin decoction were flavonoids, saponins, alkaloids and organic acids analyzed by ultra-high performance liquid chromatography-mass spectrometry (UHPLC-MS) (Ren et al. 2015). The Da-Yuan-Yin decoction water extract could decrease the ratio of lung wet-to-dry weight (W/D) and the total protein concentration in the ALI mice (Wei et al. 2017). On this basis, using the acute lung injury model, the Da-Yuan-Yin decoction polyphenol fraction (DYY-4) was obtained by pharmacological activity guidance. In this paper, the ALI mice induced by LPS-integrated network pharmacology were used to investigate the effects and mechanisms of DYY-4 on ALI.

## Materials and methods

### Preparation and component analysis of DYY-4

Chinese Medicine components of Da-Yuan-Yin, which were all planted in China, were purchased from Suzhou Tianling Traditional Chinese Medicine Beverage Co., Ltd, Jiangsu Province of China, in October 2013. Referring to Chinese Pharmacopoeia, Chinese Medicine components of Da-Yuan-Yin were authenticated by Ph.D. Chunyan Min, Suzhou Institute for Food and Drug Control, and Prof. Lili Hao, Soochow University, China. The voucher specimen (DYY-201309) is deposited in the Herbarium of Material Media, College of Pharmaceutical Science, Soochow University, China. Da-Yuan-Yin components (2000 g) were refluxed twice with water (20 L) for 2 h each time to obtain the Da-Yuan-Yin decoction water extract. After filtration, ethanol was added to obtain the supernatant of Da-Yuan-Yin decoction (DYY-CSL). DYY-CSL was added into the column loading-treated octadecyl silyl. DYY-4 was obtained by eluting with MeOH/H_2_O (60:40, ν/ν). The corresponding fractions of each herbal medicine (BL-4, CG-4, HP-4, HQ-4, ZM-4, BS-4, GC-4) were obtained according to the preparation of DYY-4.

DYY-4 (20.0 g) was fractionated on silica gel by CHCl_3_/MeOH (from 100:4 to 1:1, ν/ν) to yield five fractions (Fr.1-5). Fr. 1 was purified with a ODS column and eluted with MeOH/H_2_O (from 40:60 to 60:40, ν/ν), further eluted by Preparative high-performance liquid chromatography (P-HPLC) with MeOH/H_2_O (55:45, ν/ν) to obtain compound **1** (8.0 mg) and compound **2** (14.0 mg). Fr. 2 was purified with a ODS column and eluted with MeOH/H_2_O (from 40:60 to 60:40, ν/ν) to obtain two fractions (Fr.2.1-2.2), then Fr.2.1 was eluted on Sephadex LH-20 with MeOH to obtain compound **3** (12.0 mg), then Fr.2.1 was eluted on P-HPLC with MeOH/H_2_O (60:40, ν/ν) to obtain compound **4** (18.0 mg) and compound **5** (20.0 mg). Fraction 3 was purified with a ODS column and eluted with MeOH/H_2_O (from 40:60 to 60:40, ν/ν) to obtain two fractions (Fr.3.1-3.2), then Fr.3.2 was eluted by P-HPLC with MeOH/H_2_O (70:30, ν/ν) to obtain compound **6** (4.0 mg). Fraction 4 was purified by P-HPLC with MeOH/H_2_O (70:30, ν/ν) to obtain compound **7** (3.0 mg). Fraction 5 was decolourized by acetone to obtain compound **8** (7.0 mg).

Total polyphenol content of DYY-4 was measured by the Folin-Ciocalteu colourimetric method, and gallic acid was used as the standard (Roy et al. 2007). UHPLC-MS of DYY-4 was performed on Thermo Scientific™ LC-MS system (Thermo Fisher Scientific, USA) with a UPLC C18 analytical column (Thermo Hypersil Gold 2.1 mm × 100 mm, I.D. 3.0 μm) at 20 °C. The mobile phase was a mixture of water containing 0.2% formic acid (A) and methanol (B) with gradient elution (0 min: 10% B, 12 min: 40% B, 30 min: 40% B, 50 min: 50% B, 65 min: 90% B; 70 min: 95% B). The injection volume was 1 μL, and the flow rate was set at 0.4 mL/min. Mass spectra were obtained in positive mode, and the source parameters were set as follows: Capillary Temp 320 °C, Sheath Gas Flow 40 L/min, Aux Gas Flow 10 L/min, Spray Voltage 3.80, and S-lens RF Level 50.0. Data analysis was performed with Thermo Scientific™ Xcalibur™ software. Results are shown by base peak chromatogram (BPC) with *m/z* range 150–2000. The principal component analysis (PCA) of DYY-4 was processed with simca-p13.0.

### Reagents

LPS (No. 013M4029V) was purchased from Sigma-Aldrich Co., Ltd (St. Louis, MO, USA). Dexamethasone (DXM) acetate tablets (No. H33020822) were purchased from Zhejiang Xianju Pharmaceutical Co., Ltd. (Hangzhou, Zhejiang, China). Mouse TNF-α, IL-6 and IL-1β, IL-10, IL-4, IL-13 enzyme-linked immunosorbent assay ELISA (No. B1307233) kits were purchased from Shanghai Chuanfu Biotechnology Co., Ltd. (Shanghai, China). Mouse C3, C3c, C5a ELISA kits (No. E20130801A) and bicinchoninic acid (BCA, No. L08J7G15848) protein assay kit were purchased from Shanghai Yuanye Biotechnology Co., Ltd. (Shanghai, China). Mouse MPO, SOD and C5b-9, C5aR1 ELISA kits were purchased from Shanghai ZeYe Biotechnology Co., Ltd. (Shanghai, China). Polyclonal rabbit anti-human C3c complement (No. AB 204121) was purchased from Shanghai Ruiqi Biological Technology Co., Ltd. (Shanghai, China). Phospho-NF-κBp65 (Ser536) (93H1) rabbit monoclonal antibody (mAb), NF-κB p65 (D14E12) XP^®^ rabbit mAb, phospho-IKKα/β (Ser176/180) (16A6) rabbit mAb and IKKβ (L570) antibody rabbit mAb were purchased from Cell Signalling Technology Inc. (Beverly, MA). GAPDH mouse mAb was purchased from China Wuhan Sanying Biotechnology Co., Ltd. (Wuhan, China). Peroxidase-conjugated Affinipure goat Anti-mouse IgG (HtL) and Peroxidase-conjugated Affinipure goat Antirabbit IgG (HtL) were purchased from China Biyuntian Biotechnology Co., Ltd. (Shanghai, China). Sheep erythrocytes were collected in Alsevers’ solution. Anti-sheep erythrocyte antibody was obtained from rabbit antiserum and kindly provided by Prof. Yunyi Zhang (Department of Pharmacology, School of Pharmacy, Fudan University, Shanghai, China). Rabbit erythrocytes were obtained from the ear vein of New Zealand white rabbits. The isotonic veronal-buffered saline (VBS^2+^) buffer contained 0.5 mmol/L Mg^2+^ and 0.15 mmol/L Ca^2+^. Heparin (sodium salt, 160 IU/mg) was purchased from Shanghai Aizite Biotech Co. Ltd. All other reagents were of the highest quality available.

### Anti-complementary activity analysis

The 1:60 diluted guinea pig serum was chosen to give submaximal lysis in the absence of complement inhibitors. Various dilutions of tested samples (200 μL) were mixed with 200 μL of guinea pig serum (Jackson Immuno Research), and 200 μL of sensitized erythrocytes (EAs) was added, then the mixture was incubated at 37 °C for 30 min. Optical density of the supernatant of the reacted mixture was measured at 405 nm (Labsystems Dragon). Results were indicated in the percentage of haemolytic inhibition (Chu et al. 2014). Inhibition of lysis (%) = 100 − 100 × (OD_sample_ − OD_sample background_) ÷ OD_100% lysis_.

### Establishment of the ALI model and preventive regimen

Male Balb/c mice, about 20–25 g, were purchased from the Centre of Experimental Animals, Soochow University. All applicable international, national, and institutional guidelines for the care and use of animals were followed. All animal studies were approved by the Animal Ethics and Research Committee of Soochow University (NO. 2016121512). The mice were kept in a specific laboratory at 24 ± 1 °C and 60% relative humidity and received food and water *ad libitum*. Before experimentation, the mice were allowed to adapt to the experimental environment for 3 days.

DYY-4 was ground and suspended in distilled water containing 0.5% sodium carboxymethyl cellulose (CMC-Na) for administration to mice. The mice were divided into seven groups (each group, *n* = 10): control group, DYY-4 group (mice were treated only with DYY-4 at 60 mg/kg), LPS group (the mice were treated only with LPS at 2 mg/kg to induce ALI), LPS + DYY-4 group (the mice were treated with DYY-4 at 15, 30, and 60 mg/kg after LPS challenge, respectively) and LPS + DXM group (the mice were treated with DXM at 5 mg/kg after LPS challenge). The mice were anesthetized with 20% urethane (4 mL/kg). LPS (2 mg/kg) was instilled intratracheally (i.t.) to induce ALI (Wei et al. 2017; Huang et al. 2019). Half an hour after LPS challenge, the first administration was given, and the second administration was carried out to enhance the therapeutic effect 1 h later. Twenty-four h after LPS challenge, the blood samples were collected from eyeball blood with EDTA, then the mice were sacrificed by cervical dislocation, and the lungs were collected.

### Lung W/D ratio, protein content, cytokines, complement, MPO, and SOD analysis

Twenty-four hours after LPS challenge, the left lung was excised, blotted dry, and weighed to obtain the ‘wet’ weight, and then placed in an oven at 80 °C for 48 h to obtain the ‘dry’ weight. The ratio of the wet lung to the dry lung was calculated to assess tissue edoema. The right lung of mice was used to collect BALF, which was lavaged three times with 0.8 mL of autoclaved normal saline. The protein content and the levels of cytokines [tumor necrosis factor-α (TNF-α), interleukin-6 (IL-6), interleukin-1 β (IL-1β), interleukin-10 (IL-10), interleukin-4 (IL-4) and interleukin-13 (IL-13)] in the supernatants of the BALF were analyzed by enzyme-linked immunosorbent assay (ELISA) kits according to the manufacturer’s instructions. Blood samples were coagulated at room temperature for 10 min, and then centrifuged (4 °C, 1400 *g,* 20 min), and its supernatants (serum) were stored at −80 °C for subsequent analysis. The levels of complement (C3, C3c, C5a, C5aR1, and C5b-9) in serum were quantified by the ELISA kit according to the manufacturer’s instructions strictly. The superior lobe of the right lung was homogenized to measure the levels of MPO and SOD by ELISA kits.

### Quantitative real-time PCR

Trizol (Sangon Biotech, China) was used to extract the total mRNA in the lung, while the purity of mRNA preparation was checked by measuring the absorbance ratio at 260/280 nm. The experimental processes included 500 ng (1 μL) of total mRNA, 1 μL gDNA Purge, 2 × NovoScript 1^st^ Strand cDNA Synthesis SuperMix 10 μL, and Rnase Free Water 8 μL, respectively. For the synthesis of cDNA a transcription kit (NovoScript^®^ Plus All-in-one 1-st-Strand cDNA Synthesis SuperMix (gDNA Purge)) was used. 40 different cycling programs were established using real-time PCR which included run at 50 °C for 25 min and 75 °C for 5 min, hence at the end each analysis was run from 50-75 °C, respectively. Target cDNA levels were determined with the help of ΔCt method. Fold of suppression Expiration = 2^Ct(control)-Ct(treatment)^. The primer sequences were designed as follows: iNOS: forward 5′-AGCAACTACTGCTGGTGGTG-3′; reverse 5′-TCTTCAGAGTCTGCCCATTG-3′; COX-2: forward 5′-CAGTTTATGTCTGTCCAGAGTTTC-3′; reverse 5′-CCAGCACTTCACCCATCAGTT-3′; β–action: forward 5′-CCCATCTATGAGGGTTACGC-3′; reverse 5′-TTTAATGTCACGCACGATTTC-3′. RT-qPCR was performed using the NovoScript^®^ Plus All-in-one 1-st-Strand cDNA Synthesis SuperMix (gDNA Purge) kit (Novoprotein).

### Western blot analysis

Protein samples of lung tissue were prepared in RIPA lysis buffer with freshly added protease and phosphatase inhibitors (Biomake, Shanghai, China) and then centrifuged for 10 min at 4 °C and 12000 *g*. Protein was separated by SDS/PAGE and transferred onto PVDF membrane (BIO-RAD Trans-Blot®Turbo 1704150, USA). Membranes were probed with the following antibodies: Phospho-NF-κBp65 (Ser536) (93H1) rabbit mAb, NF-κB p65 (D14E12) XP^®^ rabbit mAb, avidt-IKKα/β (Ser176/180) (16A6) rabbit mAb, IKKβ (L570) antibody rabbit mAb, IKKα antibody rabbit and GAPDH mouse mAb. The protein bands were measured using an ECL detection system (Tanon, Shanghai, China), and semi-quantified using Image J software. The Western blot analysis results were normalized to the band intensity of GAPDH.

### Histological studies of lung

Histological studies of the inferior lobe of right lung tissue were carried out by paraffin sections stained with haematoxylin and eosin (H&E). The 5 μm sections were deparaffinized, rehydrated, and incubated with rabbit anti-human C3c overnight at 4 °C. The complement deposits of the slices were visualized by using the chromogenic substrate solution 3, 3′-diaminobenzidine. All slides were constantly observed and imaged at an original magnification of 400× (Wei et al. 2017; Huang et al. 2019).

### Statistical analysis

The obtained data were analyzed using SPSS 19.0 statistical software. To analyze the differences, a One-way analysis of variance (ANOVA) was applied, while *post hoc* comparisons were accomplished by Fisher’s PLSD.

### The network pharmacological strategy of DYY-4 for alleviating ALI

**Figure 1.**
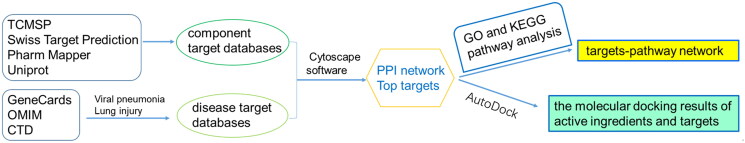
The network pharmacological strategy of DYY-4 for alleviating ALI.

Network pharmacology was used to predict the mechanisms and potential targets of DYY-4 for alleviating ALI (Figure 1). We used the TCMSP databases, Swiss Target Prediction (https://www.swisstargetprediction.ch), Pharm Mapper (http://www.lilab-ecust.cn/pharmmapper/) databases and the Uniprot databases (https://www.uniprot.org/) to predict potential targets of the main ingredients of DYY-4. We searched for disease-related targets by entering keywords ‘Viral pneumonia’ and ‘Lung injury’ into the GeneCards databases (https://www.genecards.org), OMIM databases (https://omim.org) and CTD databases (http://ctdbase.org/). The Venn diagram was used to map drug targets and disease targets to obtain the potential targets of compounds. The String databases (https://www.string-db.org/) was used to analyze the protein interaction relationship of the therapeutic targets, and the species was limited to ‘*Homo sapiens*’ (Combined score ≥ 0.9). The protein interaction relationship was imported into Cytoscape software 3.72 to construct protein-protein interaction (PPI) network. The ‘Network Analyzer’ tool was used to calculate the degree value of targets and the targets larger than the corresponding median values were recognized as crucial targets (Jin et al. 2021).

The crucial targets were used for Gene Ontology (GO) and Kyoto Encyclopaedia of Genes and Genomes (KEGG) enrichment pathway analyses by using the David databases (https://david.ncifcrf.gov/). The pathway terms with *p* < 0.05 were mapped to the KEGG human diseases in order to obtain the pneumonia-related pathways and construct component-core targets-pathway networks. We used PDB (http://www.rcsb.org/) to obtain the structural data of the top ten protein targets of DYY-4 based on PPI network analysis. The ten proteins were modified using AutoDock Tools for hydrogenation, ligands and water removal. Finally, we used AutoDock Vina 1.5.6 to perform molecular docking of the ten proteins with the compounds. We selected the receptors and ligands with strong binding energy and generated three-dimensional graphs in PyMOL to analyze their interactions.

## Results

### The components analysis of DYY-4

**Figure 2.**
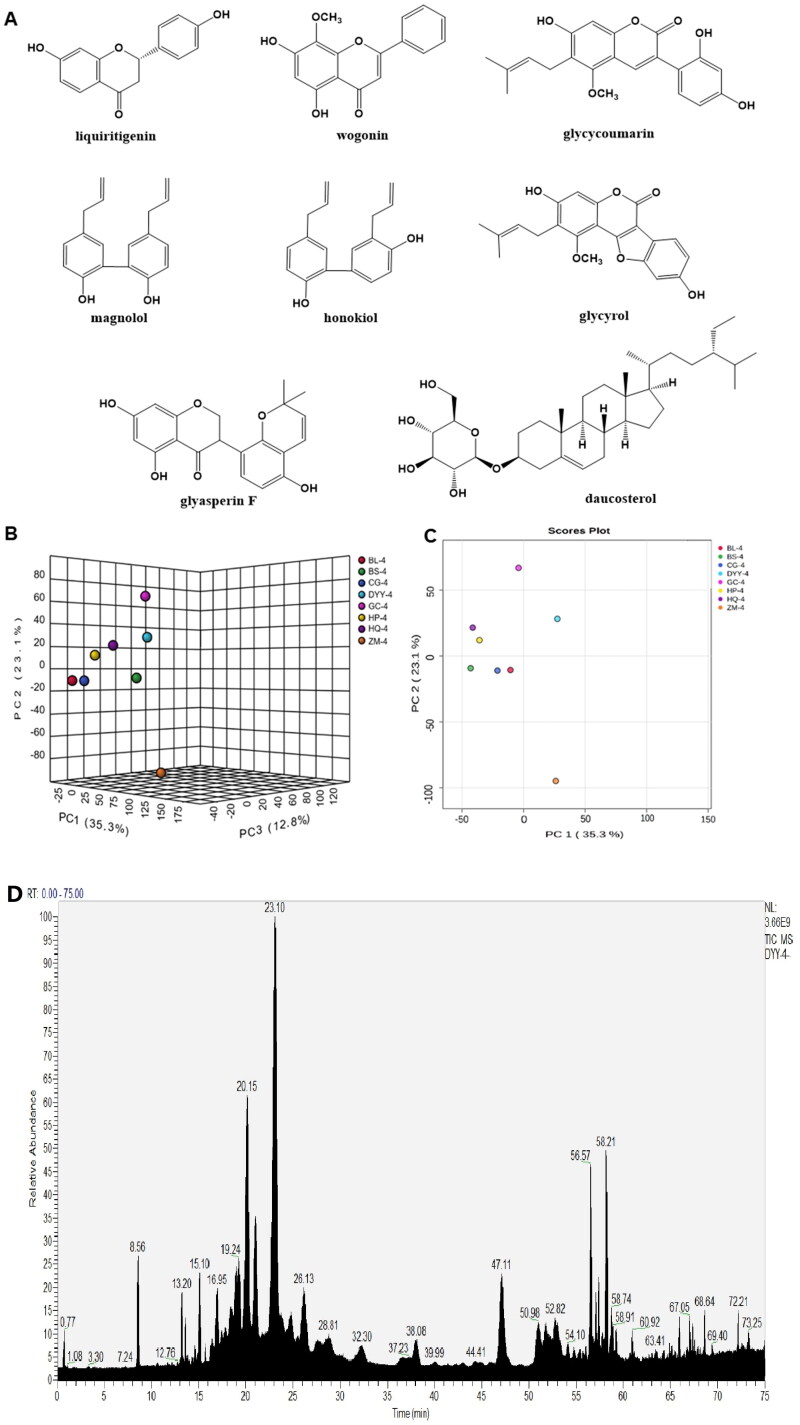
Components of DYY-4. The structures of compounds (A), score scatter 3D plot of PCA (B), score scatter plot of PCA (C) and the total ion chromatogram of DYY-4 (D).

Data of the compounds were compared with the reported literature (McKee et al. 1997; Cui et al. 2008; Lu et al. 2012; Wang et al. 2012; Jin et al. 2021), which identified and confirmed compounds **1**-**8** as liquiritigenin, wogonin, glycycoumarin, magnolol, honokiol, glycyrol, glyasperin F and daucosterol (Figure 2(A)), respectively. Compound **1** was identified as liquiritigenin, EI-MS *m/z*: 257.08 [M + H]^+^. ^1^H-NMR (600 MHz, CD_3_OD) *δ*: 7.72 (1 H, d, *J* = 9.0 Hz, H-5), 7.31 (2 H, d, *J* = 8.4 Hz, H-2′, 6′), 6.80 (2 H, d, *J* = 8.4 Hz, H-3′, 5′), 6.48 (1 H, dd, *J* = 9.0, 2.0 Hz, H-6), 6.34 (1 H, d, *J* = 2.0 Hz, H-8), 5.37 (1 H, dd, *J* = 3.0, 13.2 Hz, H-2), 3.04 (1 H, dd, *J* = 13.2, 16.8 Hz, H-3*β*), 2.68 (1 H, dd, *J* = 3.0, 16.8 Hz, H-3*α*); ^13^C-NMR (150 MHz, CD_3_OD) *δ*: 192.1 (C-4), 165.4 (C-9), 164.1 (C-7), 157.5 (C-4′), 129.9 (C-1′), 128.4 (C-5), 127.6 (C-2′, 6′), 114.9 (C-3′, 5′), 113.5 (C-10), 110.3 (C-6), 102.4 (C-8), 79.2 (C-2), 43.5 (C-3). Compound **2** was identified as wogonin, EI-MS *m/z*: 285.07 [M + H]^+^. ^1^H-NMR (600 MHz, CD_3_OD) *δ*: 8.02 (2 H, m, H-2′, 6′), 7.58 (3 H, m, H-3′, 4′, 5′), 6.76 (1 H, s, H-6), 6.29 (1 H, s, H-3), 3.93 (3 H, s, OCH_3_); ^13^C-NMR (150 MHz, CD_3_OD) *δ*: 182.6 (C-4), 164.0 (C-5), 157.7 (C-7), 156.9 (C-2), 149.9 (C-9), 132.5 (C-4′), 131.7 (C-1′), 128.9 (C-2′, 6′), 128.0 (C-8), 126.0 (C-3′, 5′), 104.5 (C-3), 103.9 (C-10), 99.0 (C-6), 60.6 (OCH_3_). Compound **3** was identified as glycycoumarin, EI-MS *m/z*: 369.13 [M + H]^+^. ^1^H-NMR (600 MHz, CD_3_OD) *δ*: 7.96 (1 H, s, H-4), 7.14 (1 H, d, *J* = 8.4 Hz, H-6′), 6.58 (1 H, s, H-8), 6.38 (1 H, dd, *J* = 8.4, 2.4 Hz, H-5′), 6.36 (1 H, d, *J* = 2.4 Hz, H-3′), 5.22 (1 H, t, *J* = 7.2 Hz, (CH_3_)_2_C=CHCH_2_), 3.83 (3 H, s, OCH_3_), 3.36 (2 H, d, *J* = 7.2 Hz, (CH_3_)_2_C = CHCH_2_), 1.78 (3 H, s, (CH_3_)_2_C = CHCH_2_), 1.68 (3 H, s, (CH_3_)_2_C = CHCH_2_); ^13^C-NMR (150 MHz, CD_3_OD) *δ*: 162.4 (C-2), 160.3 (C-4′), 158.5 (C-2′), 155.9 (C-7), 155.7 (C-9), 153.4 (C-5), 137.9 (C-4), 131.3 (C-6′), 130.8 (C-3″), 122.5 (C-3), 120.3 (C-2″), 119.4 (C-6), 114.1 (C-1′), 106.7 (C-5′), 106.5 (C-10), 102.6 (C-3′), 97.7 (C-8), 62.2 (OCH_3_), 24.5 (C-5″), 22.2 (C-1″), 16.6 (C-4″). Compound **4** was identified as magnolol, EI-MS *m/z*: 267.13 [M + H]^+^. ^1^H-NMR (400 MHz, DMSO-*d*_6_) *δ*: 7.00 (2 H, m, H-6, 6′), 6.99 (2 H, m, H-4, 4′), 6.87 (2 H, m, H-3, 3′), 6.00 (2 H, H-8, 8′), 5.10 (4 H, m, H-9, 9′), 3.32 (4 H, m, H-7, 7′); ^13^C-NMR (100 MHz, DMSO-*d*_6_) *δ*: 152.8 (C-2, 2′), 138.3 (C-8, 8′), 131.3 (C-5, 5′), 130.0 (C-4, 4′), 127.9 (C-6, 6′), 125.9 (C-1, 1′), 115.8 (C-3, 3′), 115.2 (C-9, 9′), 38.8 (C-7, 7′). Compound **5** was identified as honokiol, mp 88.0-90.0 °C. EI-MS *m/z*: 267.13 [M + H]^+^. ^1^H-NMR (400 MHz, DMSO-*d*_6_) *δ*: 7.26 (1 H, d, *J* = 2.0 Hz, H-2′), 7.24 (1 H, m, H-4), 7.02 (1 H, d, *J* = 2.0 Hz, H-6), 6.92 (1 H, dd, *J* = 2.0, 8.2 Hz, H-6′), 6.89 (2 H, d, *J* = 6.0 Hz, H-3, 5′), 6.00 (2 H, m, H-8, 8′), 5.12 (1 H, dd, *J* = 1.8, 16.8 Hz, H-9′ _b_), 5.05 (1 H, d, *J* = 10.0 Hz, H-9′_a_), 5.08 (1 H, dd, *J* = 1.8, 17.0 Hz, H-9 _b_), 5.03 (1 H, d, *J* = 10.0 Hz, H-9_a_), 3.37 (2 H, d, *J* = 6.4 Hz, H-7), 3.31 (2 H, d, *J* = 6.4 Hz, H-7′); ^13^C-NMR (100 MHz, DMSO-*d*_6_) *δ*: 153.8 (C-2), 152.4 (C-4′), 138.3 (C-8), 137.2 (C-8′), 130.4 (C-5), 130.1 (C-6), 129.9 (C-4), 129.4 (C-2′), 127.8 (C-1′), 127.8 (C-6′), 127.4 (C-3′), 125.2 (C-1), 115.9 (C-9′), 115.2 (C-9), 115.2 (C-3), 114.4 (C-5′), 38.8 (C-7), 33.9 (C-7′). Compound **6** was identified as glycyrol. EI-MS *m/z*: 367.11 [M + H]^+^. ^1^H-NMR (400 MHz, DMSO-*d*_6_) *δ*: 10.81 (1 H, s), 10.02 (1 H, s), 7.72 (1 H, d, *J* = 8.4 Hz, H-6′), 7.17 (1 H, d, *J* = 2.0 Hz, H-3′), 6.96 (1 H, dd, *J* = 8.0, 2.0 Hz, H-5′), 6.78 (1 H, s, H-8), 5.21 (1 H, t, *J* = 6.8 Hz, (CH_3_)_2_C=CHCH_2_), 3.90 (3 H, s, -OCH_3_), 3.33 (2 H, d, *J* = 6.8 Hz, (CH_3_)_2_C = CHCH_2_), 1.77 (3 H, s, (CH_3_)_2_C = CHCH_2_), 1.65 (3 H, s, (CH_3_)_2_C = CHCH_2_);^13^C-NMR (100 MHz, DMSO-*d*_6_) *δ*: 159.5 (C-7), 158.2 (C-4), 157.5 (C-2), 156.9 (C-2′), 156.1 (C-4′), 153.8 (C-5), 152.9 (C-9), 130.9 (C-6′), 122.4 (C-3″), 120.5 (C-3), 119.7 (C-2″), 114.3 (C-6), 114.0 (C-1′), 102.2 (C-5′), 99.7 (C-10), 99.2 (C-3′), 98.5 (C-8), 62.4 (OCH_3_), 25.5 (C-5″), 22.0 (C-1″), 17.7 (C-4″). Compound **7** was identified as glyasperin F. EI-MS *m/z*: 355.11 [M + H]^+^. ^1^H-NMR (600 MHz, CD_3_OD) *δ*: 6.87 (1 H, d, *J* = 8.4 Hz, H-6′), 6.65 (1 H, d, *J* = 10.2 Hz, H-7′), 6.31 (1 H, d, *J* = 8.4 Hz, H-5′), 5.89 (1 H, d, *J* = 2.0 Hz, H-6), 5.88 (1 H, d, *J* = 2.0 Hz, H-8), 5.65 (1 H, d, *J* = 10.2 Hz, H-8′), 4.58 (1 H, dd, *J* = 11.2, 10.8 Hz, H-2), 4.44 (1 H, dd, *J* = 4.8, 10.8 Hz, H-2), 4.20 (1 H, dd, *J* = 4.8, 11.2 Hz, H-3), 1.38 (6 H, s, H-10′, 11′); ^13^C-NMR (150 MHz, CD_3_OD) *δ*: 197.7 (C-4), 166.9 (C-7), 164.4 (C-5), 163.7 (C-9), 153.3 (C-4′), 150.7 (C-2′), 129.2 (C-6′), 129.0 (C-8′), 116.5 (C-7′), 115.5 (C-1′), 110.9 (C-3′), 108.4 (C-5′), 102.1 (C-10), 95.7 (C-6), 94.6 (C-8), 75.0 (C-9′), 70.0 (C-2), 46.7 (C-3), 26.4 (C-10′, 11′). Compound **8** was identified as daucosterol which was confirmed by TLC comparison with an authentic sample.

Principal component analysis (PCA) was performed on mass spectrum data of DYY-4 and each herbal medicine (BL-4, CG-4, HP-4, HQ-4, ZM-4, BS-4, GC-4). Chemical components of DYY-4 mainly came from HP-4, HQ-4, and CG-4 (Figure 2(B, C)). Moreover, the content of total polyphenols in DYY-4 was found to be 13.0%.

### Anti-complementary activity of DYY-4

The anti-complementary activity of DYY-4, obtained in 50% haemolysis inhibition concentrations (CH_50_), was 0.051 ± 0.011 mg/mL and 0.063 ± 0.010 mg/mL for heparin, respectively.

### Effects of DYY-4 on lung W/D ratio, total protein concentration, and the levels of MPO and SOD

**Figure 3.**
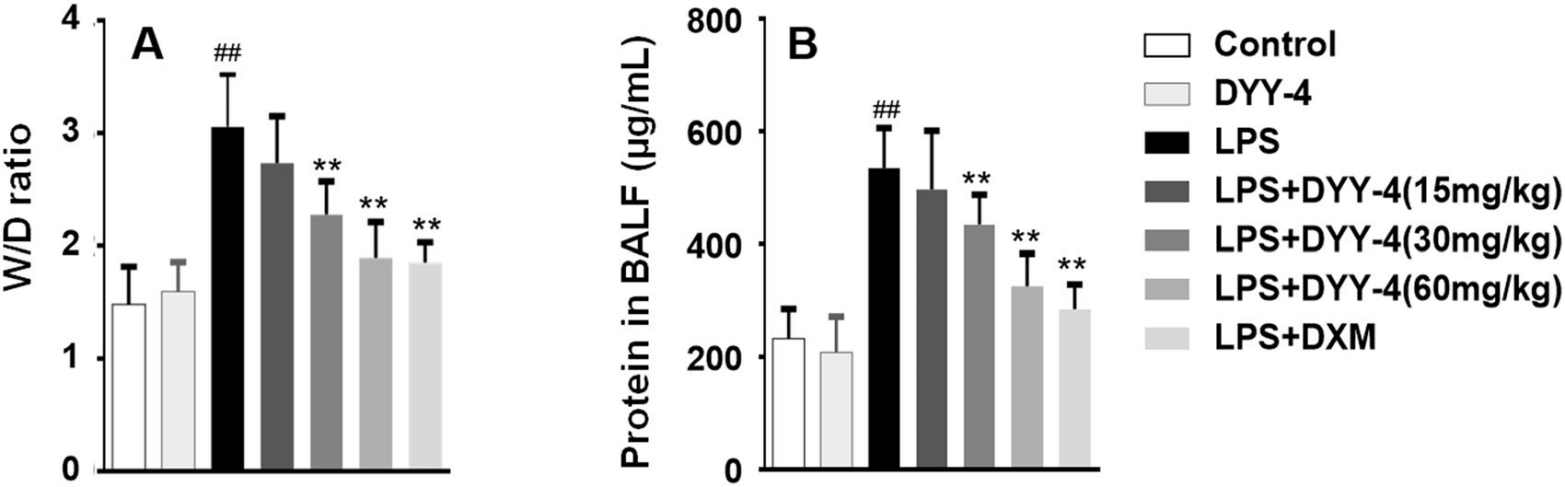
DYY-4 reduced the lung W/D ratio and total protein concentration in ALI mice. LPS (2 mg/kg) was instilled intratracheally (i.t.) to induce lung injury, half an hour after LPS challenge, DYY-4 was given to each administration group, and the second administration was carried out 1 h later. Twenty-four h after LPS challenge, lung W/D ratio (A) and protein in BALF (B) were determined. Data expressed as means ± S.D. (*n* = 10); ^##^*p* < 0.01 compared with control, **p < 0.01 compared with ALI model group.

As shown in Figure 3, compared with vehicle-treated ALI model group, DYY-4 at 30 and 60 mg/kg significantly decreased the lung W/D ratio and total protein concentration in BALF (*p* < 0.01). Similarly, DYY-4 at 60 mg/kg greatly decreased the level of MPO and increased the level of SOD (*p* < 0.01), as shown in Table 2.

**Table 2.** Effects of DYY-4 on the levels of inflammatory factors, complements and antioxidant.

	Control	LPS (2 mg/kg)	LPS + DYY-4 (60 mg/kg)	LPS + DXM (5 mg/kg)
TNF-α/IL-10 ratio	0.737 ± 0.142	3.141 ± 0.233^##^	0.996 ± 0.068**	0.595 ± 0.142**
iNOS mRNA 2(-△Ct)	0.92 ± 0.025	5.14 ± 0.035^##^	3.56 ± 0.007**	1.25 ± 0.010**
COX-2 mRNA 2(-△Ct)	0.97 ± 0.016	1.98 ± 0.021^##^	1.57 ± 0.027*	1.17 ± 0.030**
C5b-9 (pg/mL)	146.18 ± 8.6	164.35 ± 5.1^##^	157.95 ± 6.2*	134.86 ± 16.3**
C5aR1 (pg/mL)	139.31 ± 14.3	165.28 ± 4.7^##^	150.78 ± 17.6*	144.15 ± 13.1**
MPO (pg/mL)	168.92 ± 12.7	260.27 ± 32.1^##^	210.15 ± 13.3**	192.19 ± 12.1**
SOD (pg/mL)	268.56 ± 28.1	173.88 ± 25.9^##^	218.46 ± 19.6**	234.04 ± 23.9**

2 mg/kg LPS was instilled intratracheally (i.t.) to induce lung injury, half an hour after LPS challenge, DYY-4 was given to each administration group, and the second administration was carried out one hour later. Data expressed as means ± S.D. (*n* = 5); ^##^*p* < 0.01 compared with control, **p < 0.01 compared with ALI model group.

### Effects of DYY-4 on the level of inflammatory factors, the expression of iNOS, and COX-2 mRNA

**Figure 4.**
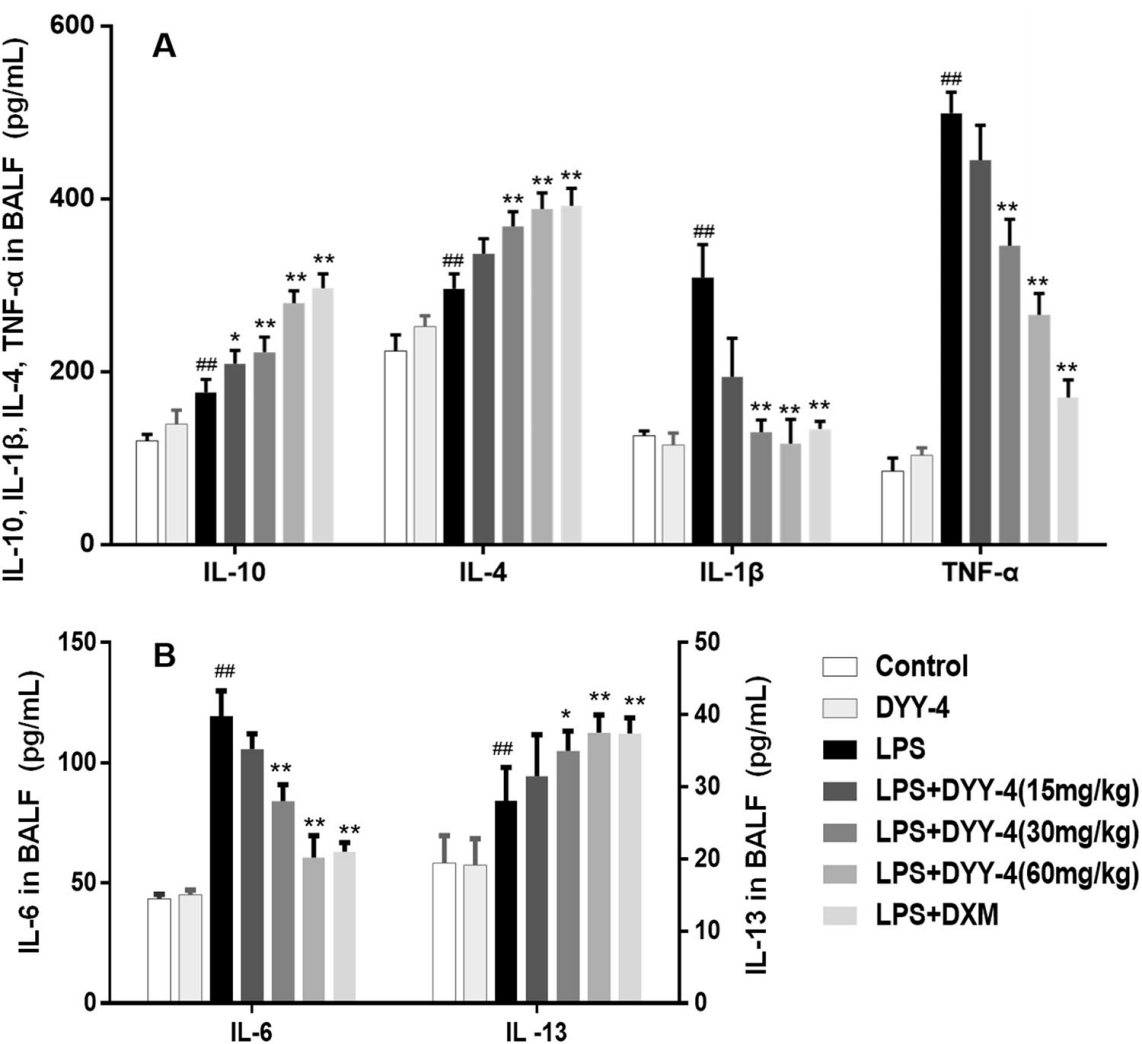
DYY-4 regulated the levels of TNF-α, IL-6 and IL-1β, IL-4, IL-13 and IL-10 in ALI mice. LPS (2 mg/kg) was instilled intratracheally (i.t.) to induce lung injury, half an hour after LPS challenge, DYY-4 was given to each administration group, and the second administration was carried out 1 h later. Twenty-four h after LPS challenge, the levels of inflammatory cytokines IL-4, IL-10, IL-1β and TNF-α (A), IL-6 and IL-13 (B) were determined. Data expressed as means ± S.D. (*n* = 10); ^##^*p* < 0.01 compared with control, *p < 0.05 and **p < 0.01 compared with ALI model group.

Compared with vehicle-treated ALI model group, DYY-4 at 30 and 60 mg/kg markedly lowered the levels of TNF-α, IL-6, and IL-1β (*p* < 0.01) depicted in Figure 4 respectively. Correspondingly, the potency of DYY-4 at 30, 60 mg/kg was observed significantly to increase the levels of IL-4 and IL-10 (*p* < 0.01), as shown in Figure 4(A). DYY-4 at 60 mg/kg markedly increased the level of IL-13 (*p* < 0.01) in BALF, while at the same concentration, the ratio of TNF-α/IL-10 and the expression of iNOS mRNA were greatly reduced, and these results were clearly shown in Figure 4(B) and Table 2, respectively. Also, DYY-4 at 60 mg/kg noticeably reduced the expression of COX-2 mRNA (*p* < 0.05, Table 2).

### Effects of DYY-4 on the levels of C3, C3c, C5a, C5b-9, and C5aR1

**Figure 5.**
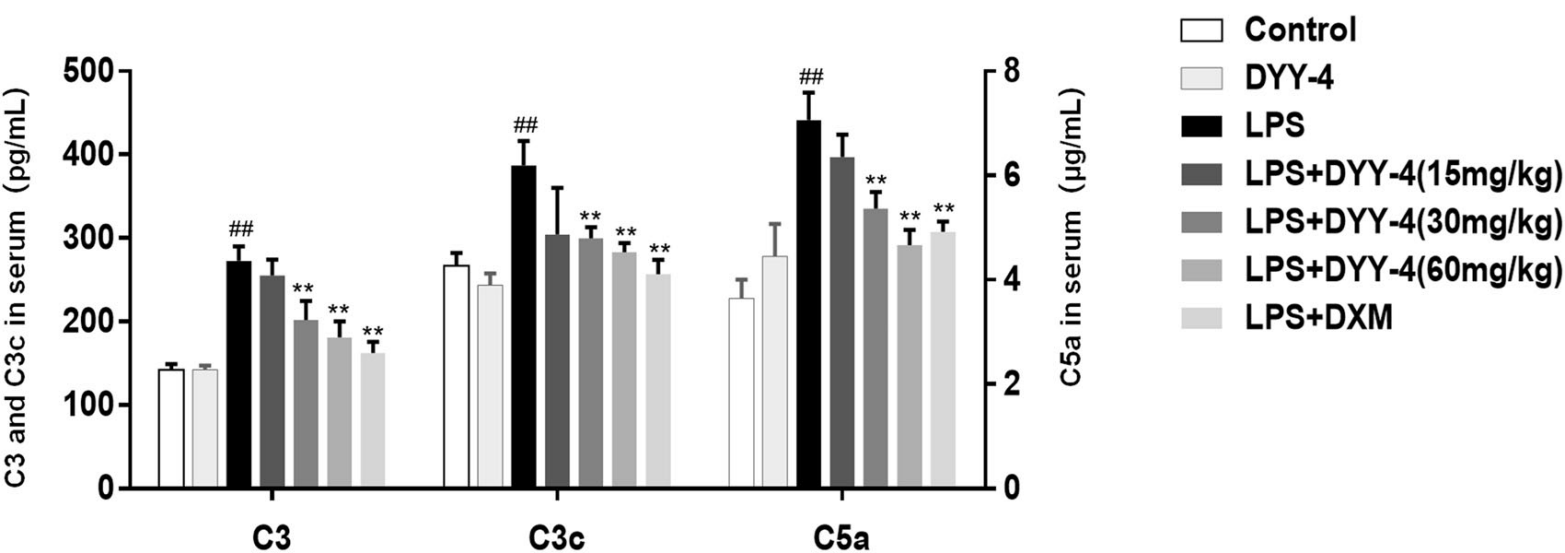
DYY-4 decreased the levels of C3, C3c and C5a in ALI mice. LPS (2 mg/kg) was instilled intratracheally (i.t.) to induce lung injury, half an hour after LPS challenge, DYY-4 was given to each administration group, and the second administration was carried out 1 h later. Twenty-four h after LPS challenge, the levels of C3, C3c and C5a in serum were determined by kit. Data expressed as means ± S.D. (*n* = 10); ^##^*p* < 0.01 compared with control, *p < 0.05 and **p < 0.01 compared with vehicle treated ALI model group.

Compared with vehicle-treated ALI model group, 30 and 60 mg/kg of DYY-4 reduced the levels of C3, C3c and C5a (*p* < 0.01, Figure 5); while 60 mg/kg of that significantly lowered the levels of C5b-9 and C5aR1 (*p* < 0.05, Table 2).

### Effects of DYY-4 on the levels of NF-κB and IKK

**Figure 6.**
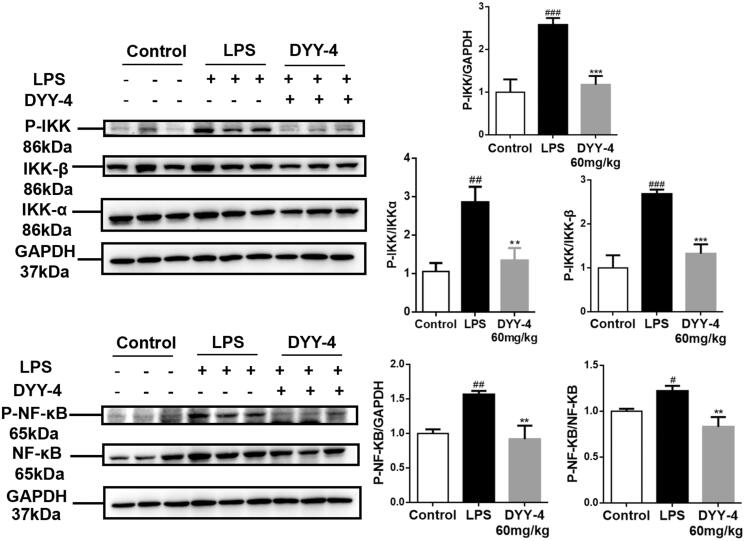
DYY-4 inhibited IKK/NF-κB inflammasome pathway in ALI mice. LPS (2 mg/kg) was instilled intratracheally (i.t.) to induce lung injury, half an hour after LPS challenge, DYY-4 was given to each administration group, and the second administration was carried out 1 h later. Twenty-four h after LPS challenge, western blot analysis was performed to detect the protein levels of IKKα, IKK-β, p-IKKα/β, NF-κB p-p65 and NF-κB p65 in lung tissue. Data expressed as means ± S.D. (*n* = 3); ^#^*p* < 0.05, ^##^*p* < 0.01 and ^###^*p* < 0.001 compared with control, **p < 0.01 and ***p < 0.001 compared with vehicle treated ALI model group.

Western blot results (Figure 6) showed that the nuclear p65 and p-p65 expression in the ALI mice were increased compared to the control group (*p* < 0.05). However, DYY-4 at 60 mg/kg effectively reversed this trend compared to the LPS group (*p* < 0.01). In the ALI mice, the protein levels of both IKK and p-IKK showed pronounced upregulation in comparison to the control group (*p* < 0.01), but DYY-4 at 60 mg/kg significantly reduced expressions of IKK and p-IKK comparison to the LPS group (*p* < 0.01, Figure 6).

### Effects of DYY-4 on lung histology

**Figure 7.**
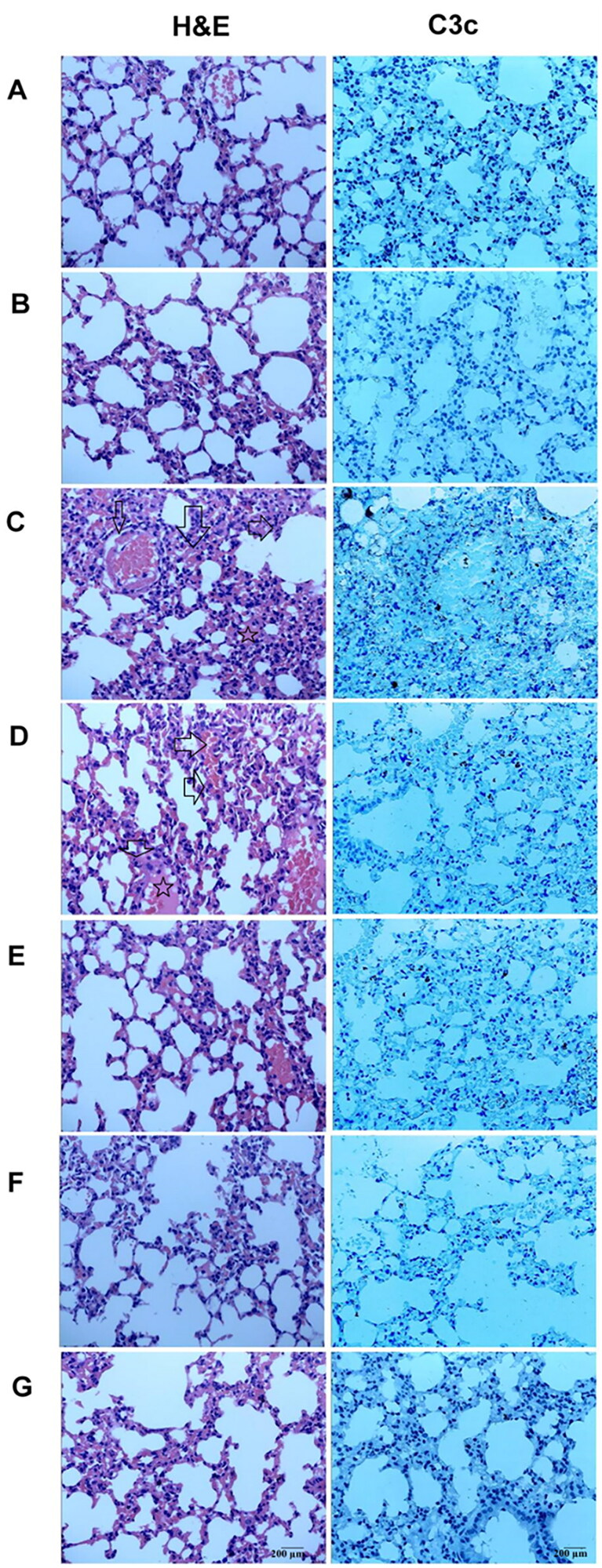
DYY-4 ameliorated the pulmonary injury in ALI mice. Lungs from each group were processed for histological evaluation at 24 h after LPS challenge: Section of control (A) and DYY-4 (B) groups mice: normal lung tissue sections (H&E and complement). Section of the LPS-induced ALI model (C) group mice: note increased alveolar wall thickness, inflammatory cells aggregation (arrows and five-pointed star), pulmonary haemorrhage (H&E), and the patchy dense immunoperoxidase indicative of depositions of complements (complement). Section from 15, 30, and 60 mg/kg DYY-4 treated (D, E, F, respectively) and DXM-treated (G) groups mice: note mild alveolar wall thickness, reduced inflammatory cells aggregation, little pulmonary haemorrhage (H&E) and little complement deposition (complement), (400 ×).

Histopathological changes of each group were observed by histochemical staining with H&E. Inflammatory cell infiltration, lung tissue damage, interstitial edoema, and alveolar wall thickening were observed in the ALI mice; while these lesions were not apparent in normal mice; DYY-4 at 30 and 60 mg/kg markedly ameliorated the pulmonary injury. In the ALI mice, immunohistochemistry of lung tissue sections showed a patchy dense immunoperoxidase indicative of complement C3c deposition. Complement C3c, appeared by bulk brown deposition, was mainly deposited in lung tissue. In contrast, mice in the control group had little complement deposition in lung tissue. DYY-4 at 60 mg/kg decreased the depositions of C3c, results were depicted in Figure 7.

### The network pharmacological approach of the main ingredients of DYY-4 for alleviating ALI

**Figure 8.**
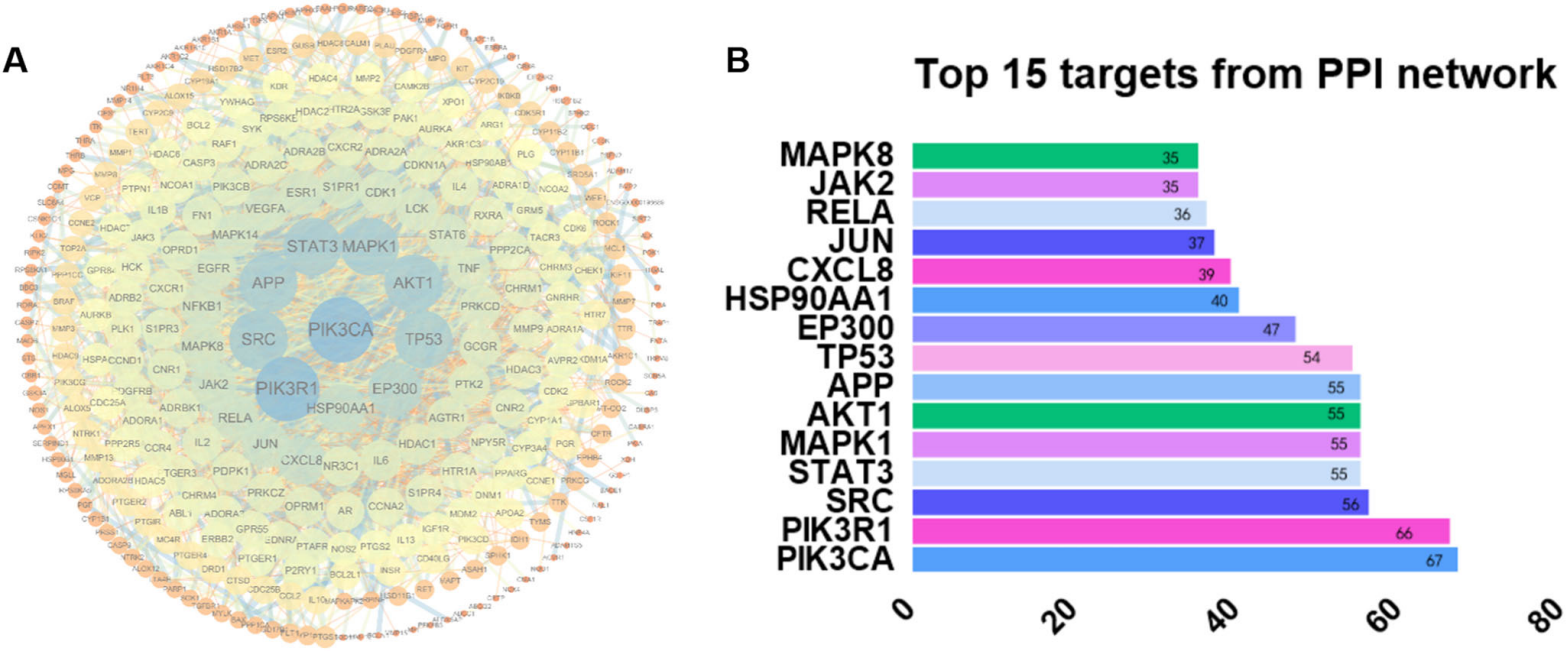
Protein-protein interaction (PPI) network and top 15 targets from the PPI network. PPI network of 74 target genes: the node colour depth and node size are directly proportional to the degree value, and the edge width and colour depth are directly proportional to the confidence of the interaction between proteins (A). Top 15 targets from PPI network (B).

TCMSP, SwissTarget, and PharmMapper databases identified 63, 350 and 71 compound-related targets, respectively. After the removal of the repeated targets, a total of 439 compound-related targets were identified. 8, 875, 70 and 7273 disease-related targets were collected by GeneCards, OMIM and CTD databases, respectively. After removing the duplicates, a total of 12,323 disease-related targets were collected. A total of 381 therapeutic targets were obtained by mapping drug targets and disease-related targets using the Venn diagram. The PPI network of therapeutic targets was constructed by Cytoscape software, as shown in Figure 8(A). After removing the isolated targets, the network consisted of 316 nodes and 2044 edges. The median of the degree of network nodes doubled was 20, and a total of 74 targets met the screening requirements as the key core targets. Among them, the 10 top degree values were PIK3CA, PIK3R1, SRC, STAT3, MAPK1, AKT1, APP, TP53, EP300, and HSP90Aa1 (Figure 8).

**Figure 9.**
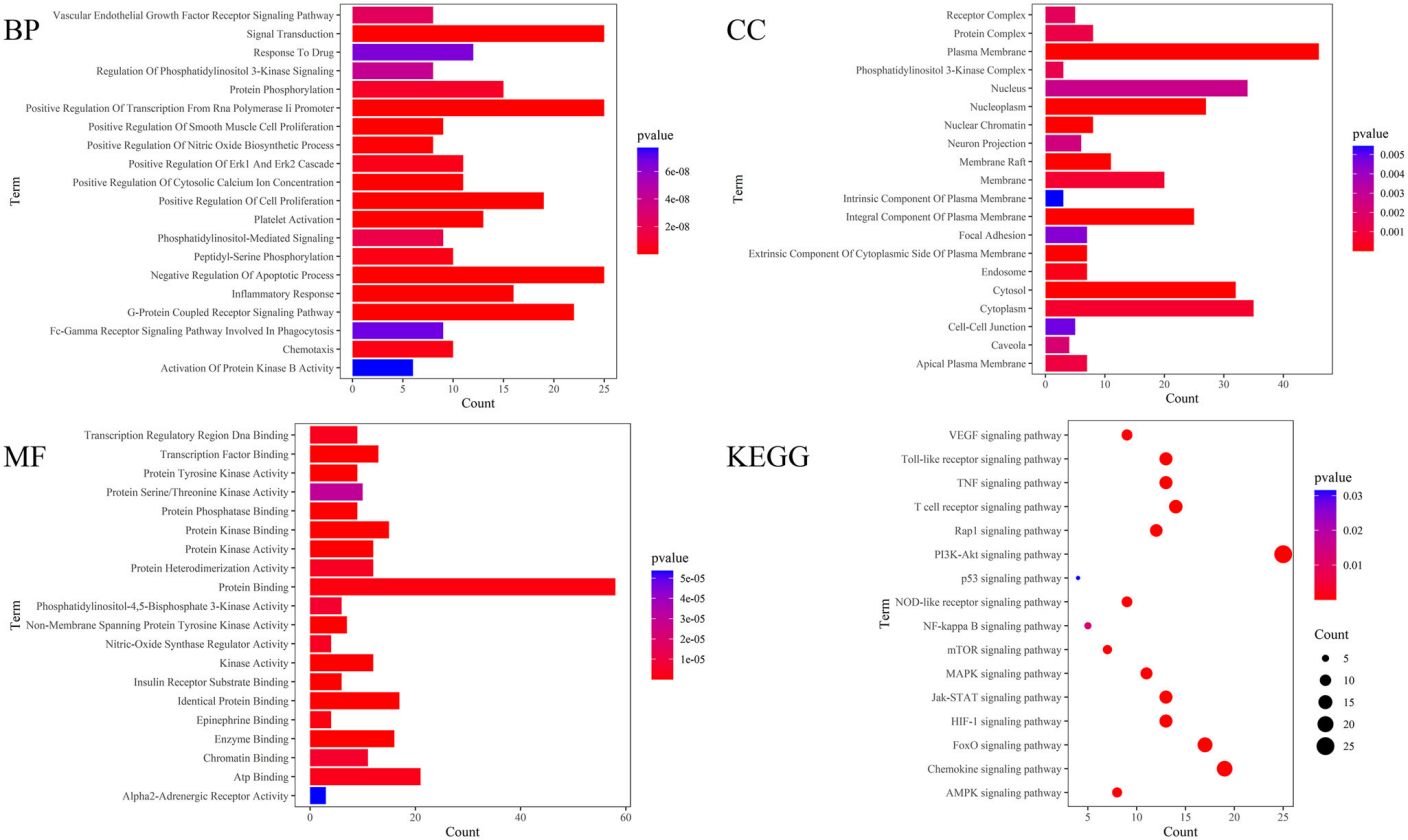
GO enrichment analysis and KEGG enrichment analysis. GO enrichment analysis, the different colour of the bar diagram indicates different P value ranges; BP: Biological process enrichment analysis; CC: Cellular component enrichment analysis; MF: Molecular function enrichment analysis. KEGG: Dotplot of KEGG analysis, the size of dots indicates the number of target genes, the different colour of dots indicates different P value ranges.

**Figure 10.**
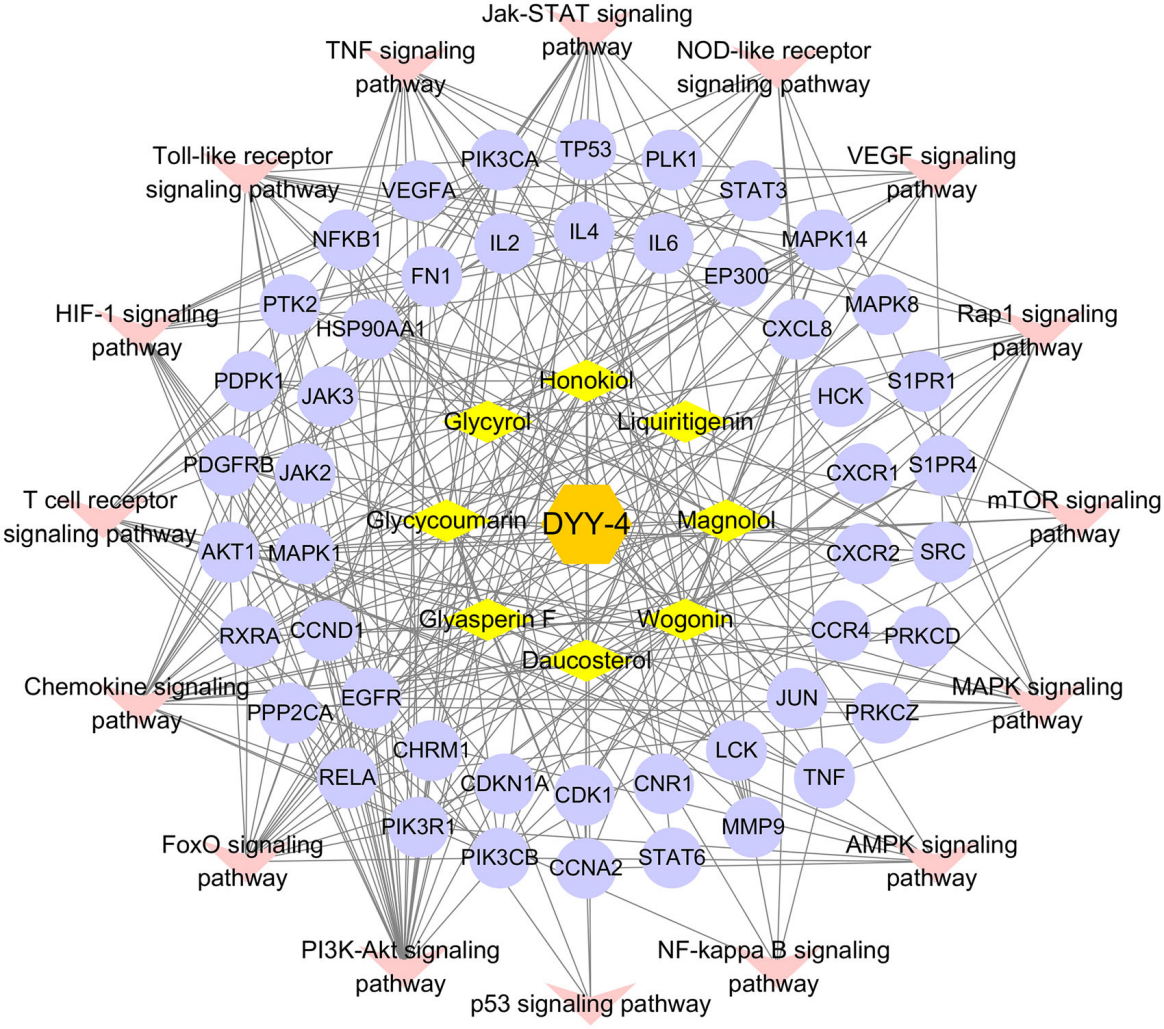
Analysis of targets-pathway network. Diamond-shaped nodes represent compounds, circular nodes represent targets, and V-shaped nodes represent KEGG pathways

The GO function and KEGG pathway enrichment analysis were performed on the therapeutic targets by using the David database, and the 20 top results in GO function analysis and KEGG results were visualized (Figure 9). A total of 406 items were enriched in GO functional enrichment analysis, including 300 items of Biological Process (BP), 40 items of Cellular Component (CC) and 66 items of Molecular Function (MF). It mainly involves Negative regulation of the apoptosis process, Positive regulation of cell proliferation, inflammatory response, positive regulation of nitric oxide biosynthesis, chemotaxis, etc. KEGG pathway enrichment analysis enriched 16 related pathways, mainly including the PI3K-Akt signalling pathway, MAPK signalling pathway, AMPK signalling pathway, p53 signalling pathway, TNF signalling pathway, Rap1 signalling pathway, NF-kappa B signalling pathway, etc. Based on KEGG pathway analysis results, a component-core target-pathway network was constructed (Figure 10). The network had a total of 73 nodes and 299 edges, including 1 drug, 8 compounds, 48 targets and 16 KEGG pathways, reflecting the multi-component, multi-target and multi-pathway characteristics of DYY-4 for alleviating ALI.

**Figure 11.**
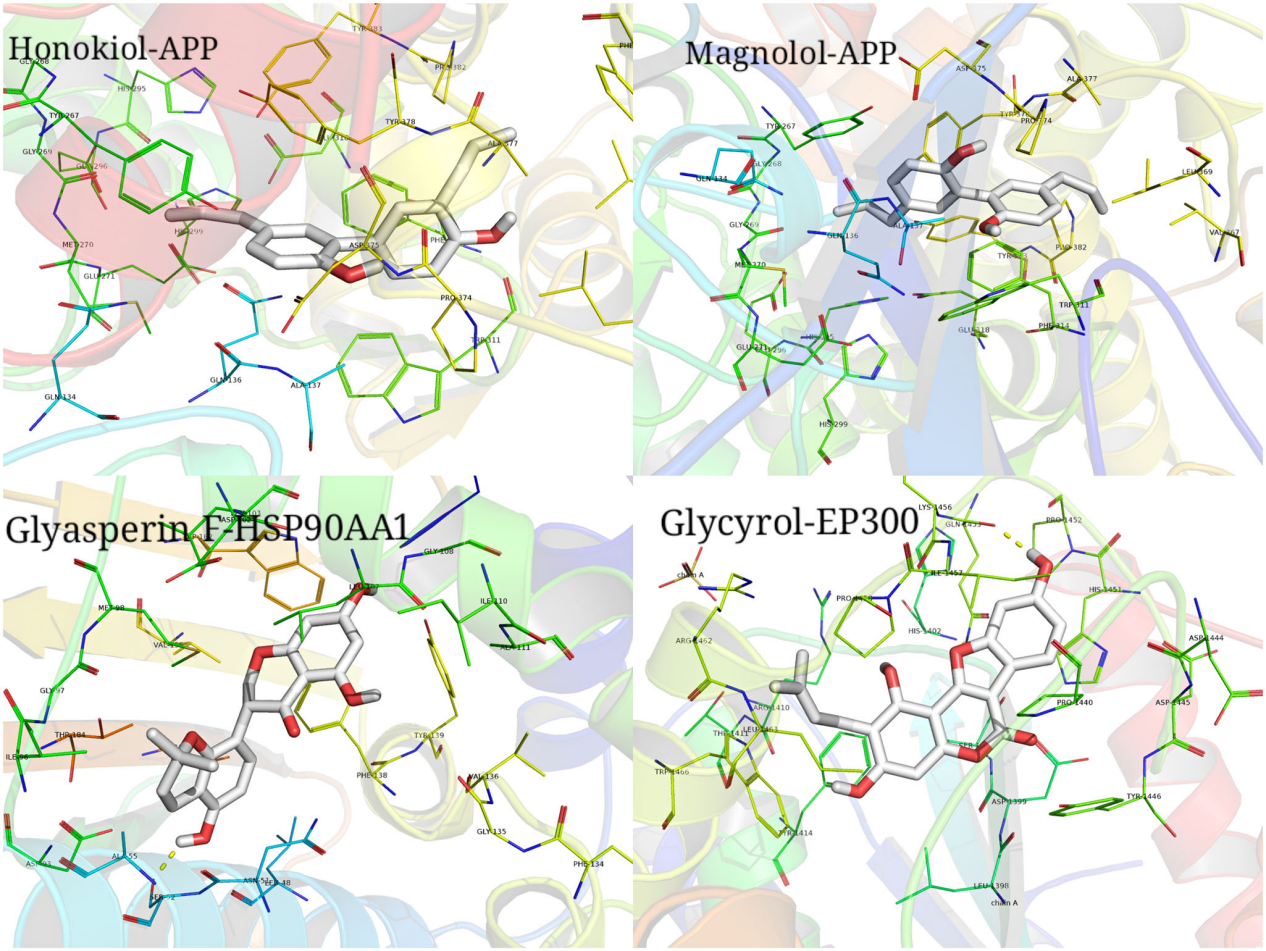
Examples of the molecular docking results of active ingredients and targets. Binding pattern between Honokiol and APP; binding pattern between Magnolol and APP; binding pattern between Glyasperin F and HSP90AA1; binding pattern between Glycyrol and EP300.

The top ten target proteins with degree values were downloaded from the PDB database, and the AutoDock Vina software was used to calculate the minimum binding energy of the compound and target protein and drew a heat map. The affinity energy is a prerequisite indicator to determine whether the ligand small molecule can effectively bind to the receptor, with lower energy values suggesting the better binding capacity for the receptor and ligand. In this study, the lowest binding energy between the compounds and the top 10 target proteins with degree values was mostly less than −5 kal/mol, indicating that they could bind well. PyMoL was used to visualize the molecular docking results (Figure 11). The target protein was closely bound to the active component by hydrogen bond, aromatic interaction and other energetic molecular interactions, further demonstrating that the active component and target protein could form a relatively stable conformation and thus combine well.

## Discussion

LPS can cause strong inflammatory and immune responses in ALI mice (Saluk-Juszczak and Wachowicz 2005; Shi et al. 2014; Wei et al. 2017; Huang et al. 2019; Pei et al. 2019; Tian et al. 2019). In this paper, the ALI model was established by intratracheal instillation of LPS, and DXM was used as the positive control, which is mostly treated with ALI/ARDS (Wei et al. 2017; Huang et al. 2019). The therapeutic effects of DYY-4 on ALI were evaluated by the ratio of lung wet-to-dry and total protein content in the BALF, which were verified by histopathologic studies. On this basis, we gradually studied the effects of DYY-4 on the levels of inflammatory factors and complements, in order to explore the pharmacological mechanism of DYY-4 against ALI.

The proinflammatory cytokines, such as TNF-α, IL-6, and IL-1β, induce and aggravate the inflammatory response (Bhatia and Moochhala 2004; Qiao et al. 2013; Shi et al. 2014; Huang et al. 2019; Pei et al. 2019; Tian et al. 2019; Um et al. 2020). IL-6 promotes neutrophils to adhere, raise, and penetrate capillaries, which eventually leads to tissue damage and pulmonary edoema. Anti-inflammatory factors, such as IL-13, IL-10, and IL-4, inhibit the synthesis of proinflammatory cytokines (Hackett et al. 2008; Wu et al. 2009; Wang et al. 2016; Pei et al. 2019; Tian et al. 2019). Similarly, IL-13 inhibits monocytes and macrophages to produce proinflammatory factors, such as IL-1, IL-6 and TNF-α (de Waal Malefyt et al. 1993). Therefore, ALI might be alleviated by inhibiting the inflammatory response and maintaining the balance of inflammatory factors. Dong-Chong-Xia-Cao extracts protected ALI *via* reducing the increased levels of TNF-α, IL-1β, and IL-6 and the expression of iNOS, COX-2 mRNA (Fu et al. 2019). Furthermore, in our previous studies, we highlighted that jaceosidin lessened ALI by lowering the levels of TNF-α, IL-6 and IL-1β, together with raising the levels of IL-4 and IL-10 (Wei et al. 2017). Similarly, the Long-Li-Ye resin eluting fraction protected ALI by downregulation of proinflammatory factor levels and TNF-α/IL-10 ratio, with upregulation of anti-inflammatory factor levels (Wei et al. 2018). The present study found that DYY-4 (30, 60 mg/kg) significantly reduced the levels of proinflammatory factors (TNF-α, IL-6, and IL-1β) and the TNF-α/IL-10 ratio, together with increasing the levels of anti-inflammatory factors (IL-4, IL-13 and IL-10). Furthermore, DYY-4 (60 mg/kg) appreciably regulated the expressions of iNOS and COX-2 mRNA, which could regulate the formation of inflammatory mediators and were often used to assess the severity of inflammation (Fu et al. 2019; Pei et al. 2019). Therefore, DYY-4 might maintain the balance of inflammatory responses in ALI.

The complement system plays critical roles in both innate and adaptive immunity by activating the classical, alternative and lectin pathways. However, complement activation also triggers potent detrimental hyperinflammatory responses that cause tissue damage and organ failure (Sarma and Ward 2011). The activation of complement can exacerbate the development of lung damage. When inflammation occurs, C3 decomposes into various complement fragments such as C3a, C3c, and C5a. These complement fragments promote the production of proinflammatory cytokines, such as TNF-α, IL-1β, and IL-6, neutrophil aggregation, and the formation of the membrane attack complex (MAC, C5b-9) to amplify the inflammatory response in ALI (Guo and Ward 2005; Bolger et al. 2007; Bosmann and Ward 2012; Wu et al. 2021). In addition, C5a binds to C5aR in alveolar macrophages and initiates downstream signalling, promotes autophagy, leads to apoptosis of alveolar macrophages, disrupts pulmonary homeostasis and then aggravates lung damage (Guo and Ward 2005; Bolger et al. 2007; Bosmann and Ward 2012; Wu et al. 2021). Thrombin-activatable fibrinolysis inhibitors protected ALI by inhibiting the level of C5a (Naito et al. 2013). Ficolin A mediated excessive complement activation exacerbated pulmonary proinflammatory responses and contributed to H1N1 influenza virus infection-induced acute lung immunopathological injury (Wu et al. 2021). In our previous studies, Jaceosidin spotlighted strong anticomplementary activity and attenuated ALI by lowering the levels of C3 and C3c in serum and reducing the deposition of C3c in lung tissue (Huang et al. 2019). Consequently, ALI might be alleviated by the downregulation of complement levels. In the present study, DYY-4 (30, 60 mg/kg) significantly decreased the levels of C3, C3c and C5a in serum, and DYY-4 (60 mg/kg) significantly decreased the levels of C5b-9 and C5aR1 in serum and deposition of C3c in lung tissue. Thus, DYY-4 alleviated ALI by decreasing the levels of complements.

Oxidative stress may cause oxidative damage to lung tissues and increase of the total content of inflammatory substances to exacerbate ALI (Wei et al. 2018; Huang et al. 2019; Pei et al. 2019). In this paper, DYY-4 (60 mg/kg) showed antioxidation by reducing the level of MPO and increasing the level of SOD to lessen ALI.

The majority of patients with COVID-19 caused by the 2019 novel coronavirus, which has now been named SARS-CoV-2 by the International Committee of Taxonomy of Viruses, have respiratory symptoms and various degrees of lung abnormalities, lymphopenia, neutrophils and inflammatory biomarkers (Marraha et al. 2020; Ouassou et al. 2020). Rapid viral replication triggers a cascade of inflammatory reactions and the high production of cytokines responsible for the accumulation of cells and fluids (Marraha et al. 2020; Ouassou et al. 2020). Therefore, the inflammatory response plays a crucial role in SARS-CoV-2-induced lung injury cases (Marraha et al. 2020; Ouassou et al. 2020). Additionally, both clinical and basic science studies suggested that uncontrolled activity of the complement system might be a central player in the pathogenesis of COVID-19 (Afzali et al. 2022). SARS-CoV-2 itself can activate the complement system either directly through the lectin pathway, the classical pathway and/or the alternative pathway. Patients with severe COVID-19 have high levels of C3, C5a and C5b-9 (Afzali et al. 2022). In the clinic, Da-Yuan-Yin decoction can alleviate the symptoms of patients with Coronavirus disease 2019 (COVID-19) in China (Headquarters for prevention and control of infected pneumonia in COVID-19, Hubei Province, China, 2020). In this paper, DYY-4 could adjust the levels of inflammatory factors, complements and antioxidant in ALI, which might be part of the pharmacodynamic mechanisms of Da-Yuan-Yin decoction in the treatment of patients with coronavirus pneumonia.

The present study showed DYY-4 protected ALI by the multi-targets, yet the correlation between the changes of inflammatory factors and complement levels was not investigated and analyzed. How DYY-4 can inhibit inflammation by reducing the levels of complements remains to be studied. Network pharmacology also failed to solve this problem. Network pharmacology can predict some signal pathways related to inflammation.

Network pharmacology is a new methodological system based on pharmacology and pharmacodynamics, which can transform the research approach of ‘one target, one drug’ into a ‘network target, multicomponent’ strategy to systematically investigate the interaction networks of compounds, targets, pathways, and diseases to elucidate the potential underlying therapeutic mechanisms of traditional Chinese medicine (Jin et al. 2021). In this paper, the network pharmacological approach reflected that the active components of DYY-4 were closely bound to the target protein by hydrogen bond, aromatic interaction and other energetic molecular interactions and DYY-4 alleviated ALI by the multi-target and multi-pathway, such as NF-κB, p38MAPK, AMPK. NF-κB signalling pathway is activated when p65 is translocated from cytosol to nucleus through IκB-α phosphorylation and subsequent degradation by activated IKK, which plays a key role in regulating the expression of the various immunomodulators such as iNOS, COX-2, IL-1β, IL-6 and TNF-α (Hu et al. 2011; Shi et al. 2014; Tian et al. 2019; Gao et al. 2020). LPS can stimulate pro-inflammatory signals that phosphorylate IκB, freeing NF-κB to translocate to the nucleus to promote pro-inflammatory expression which causes strong inflammatory responses in ALI mice (Wang et al. 2016; Wei et al. 2018; Tian et al. 2019). AMPK is a central regulator of cellular energy metabolism in eukaryotes, which can negatively regulate the inflammatory activation of macrophages by inhibiting NF-κB activity (Gao et al. 2020). In this paper, the activation of the NF-κB pathway was detected, including the phosphorylation of IKK and p65 in total protein lysates from the lung tissues. Western blot results exhibited that DYY-4 at 60 mg/kg significantly reduced the protein levels of IKK, p-IKK, the nuclear p65 and pp65. Therefore, DYY-4 attenuated ALI relating to depressing the activation of the IKK/NF-κB signal pathway.

## Conclusions

This investigation provided a comprehensive evaluation of DYY-4 attenuating ALI induced by LPS, including the levels of inflammatory factors, oxidase and complements. DYY-4 (60 mg/kg) protected lung tissues with the reduction of the ratio of lung W/D and the protein content in BALF. DYY-4 regulated the levels of inflammatory factors (TNF-α, IL-6, IL-1β, IL-4, IL-13 and IL-10) and the ratio of TNF-α/IL-10 in BALF. Meanwhile, DYY-4 (60 mg/kg) clearly reduced the levels of C3, C3c, C5a, C5aR1, and C5b-9 in serum and decreased the deposition of complement in lung tissue. DYY-4 (60 mg/kg) decreased the level of MPO and increased the level of SOD. Additionally, DYY-4 inhibited the expressions of COX-2 and iNOS mRNA. Importantly, DYY-4 (60 mg/kg) depressed the protein levels of IKK, p-IKK, the nuclear p65 and pp65. In conclusion, DYY-4 attenuated ALI induced by LPS *via* adjusting the levels of complements and inflammatory mediators, antioxidants and inhibiting the IKK/NF-κB signal pathway.
